# IL-10 Producing B Cells Dampen Protective T Cell Response and Allow *Chlamydia muridarum* Infection of the Male Genital Tract

**DOI:** 10.3389/fimmu.2019.00356

**Published:** 2019-03-01

**Authors:** Leonardo R. Sanchez, Gloria J. Godoy, Melisa Gorosito Serrán, Maria L. Breser, Facundo Fiocca Vernengo, Pablo Engel, Ruben D. Motrich, Adriana Gruppi, Virginia E. Rivero

**Affiliations:** ^1^Centro de Investigaciones en Bioquímica Clínica e Inmunología, CIBICI-CONICET, Córdoba, Argentina; ^2^Departamento de Bioquímica Clínica, Facultad de Ciencias Químicas, Universidad Nacional de Córdoba, Córdoba, Argentina; ^3^Immunology Unit, Department of Biomedical Sciences, Immunology and Neurosciences, Medical School, University of Barcelona, Barcelona, Spain

**Keywords:** *Chlamydia trachomatis*, prostatitis, regulatory B cells, male genital tract, infection, IL-10

## Abstract

A significant proportion of individuals develop chronic, persistent and recurrent genital tract infections with *Chlamydia trachomatis*, which has been attributed to the numerous strategies that the bacterium uses to subvert host immune responses. Animal chlamydia models have demonstrated that protective immune response is mediated by CD4^+^ Th1 cytokine responses. Herein, we demonstrate that early after infecting the male genital tract, *C. muridarum* triggers the production of IL-10 by splenic and lymph node cells. In addition, *C. muridarum* triggers IL-6 and TNFα secretion. Data obtained from *in vitro* and *in vivo* experiments revealed B cells as the major IL-10 contributors. Indeed, purified B cells produced high amounts of IL-10 and also exhibited enhanced expression of inhibitory molecules such as CD39, PD-L1 and PD1 after *C. muridarum* stimulation. *In vitro* experiments performed with sorted cell subsets revealed that Marginal Zone B cells were the main IL-10 producers. *In vitro* and *in vivo* studies using TLR-deficient mice indicated that TLR4 signaling pathway was essential for IL-10 production. In addition, *in vivo* treatments to neutralize IL-10 or deplete B cells indicated that IL-10 and B cells played a significant role in delaying bacterial clearance ability. Moreover, the latter was confirmed by adoptive cell transfer experiments in which the absence of IL-10-producing B cells conferred the host a greater capability to induce Th1 responses and clear the infection. Interestingly, NOD mice, which were the least efficient in clearing the infection, presented much more Marginal Zone B counts and also enhanced TLR4 expression on Marginal Zone B cells when compared to B6 and BALB/c mice. Besides, treatment with antibodies that selectively deplete Marginal Zone B cells rendered mice more capable of inducing enhanced IFNγ responses and clearing the infection. Our findings suggest that B cells play a detrimental role in *C. muridarum* infection and that activation by innate receptors like TLR4 and IL-10 production by these cells could be used by *Chlamydia* spp. as a strategy to modulate the immune response establishing chronic infections in susceptible hosts.

## Introduction

*Chlamydia trachomatis*, an obligate intracellular pathogen, is the most common cause of sexually transmitted bacterial infections worldwide (1). Despite improved surveillance and treatment initiatives, the incidence of *C. trachomatis* infection has increased dramatically over the past 30 years in both developed and developing countries (2). Approximately 75% of *C. trachomatis* infections in women and up to 50% of those in men are asymptomatic; thus, they often remain undiagnosed and/or untreated facilitating the development of chronic infections and the spread of the pathogen (1, 3). Clinical manifestations of chlamydial infections in women include urethritis, bartholinitis, cervicitis, and upper genital tract infection (including endometritis, salpingo-oophoritis, and pelvic inflammatory disease), which if left untreated can lead to severe reproductive complications (3, 4). In men, *C. trachomatis* infects urethra being a major cause of male urethritis, which usually constitutes an acute episode of an underlying chronic silent infection affecting the prostate, seminal vesicles, epididymis, and testis (5–7).

In both, female and male genital tract infections, *Chlamydia* stimulates a complex array of host innate and adaptive immune responses (6, 8–10). It has been demonstrated that innate immune receptors such as TLR4, TLR2, and others mediate the recognition of chlamydial molecular patterns. Innate immune cells rapidly recognize and limit the infection, and ultimately influence the outcome through the modulation of the adaptive immune response (11). Existing literature clearly points out CD4^+^ T cells, particularly Th1cells, as the major immune effectors for bacterial clearance in the genital tract (12–14). In addition, host regulatory pathways also become activated to limit the magnitude of excessive immunopathology (15). Although effector innate and adaptive immune responses are induced, they often fail to clear the infection or prevent subsequent re-infections (16). In fact, the specific adaptive immune response often fails to prevent re-infections, which are very frequent (3, 17). This has been attributed to several immunoevasion strategies of *C. trachomatis*, such as decreased MHC class I and CD1d expression in infected genital tract epithelial cells (18, 19). Also, *C. trachomatis* interferes with the induction of apoptosis protecting itself against the immune response (20), and modulates host cytokine production skewing immune responses (21). Noteworthy, *Chlamydia* induces the production of IL-10, a potent cytokine that can facilitate pathogen survival by negatively regulating both innate and adaptive host responses (22–24). In this regard, we recently reported higher IL-10 production and delayed bacterial clearance in NOD mice after male genital tract infection (25). Multiple cell types are capable of producing IL-10 during *Chlamydia* infection including activated macrophages, dendritic cells, keratinocytes, T and B lymphocytes (24–27). However, the contribution of IL-10 producing cells to modulate the quality, magnitude and direction of the host immune response in *C. muridarum* infection has been scarcely studied.

In the present report, comparing different mice strains and different time points we demonstrate that splenic and prostate-draining lymph node cells from infected mice produce high amounts of IL-10 in response to *C. muridarum* stimulation early after infection through the engagement of innate immune receptors. *In vitro* experiments showed that purified B cells and MZB were the main producers and suggest that IL-10 production down modulates the induction of protective Th1 responses delaying bacterial clearance.

## Materials and Methods

### *Chlamydia* Strain

*Chlamydia muridarum* Weiss strain was kindly supplied by K. H. Ramsey (USA) and propagated in LCCMK2 cells as previously described (25, 28). Briefly, cells were grown in RPMI-1640 medium supplemented with 20 μg/mL of gentamicin, 5% FBS, at 37°C and 5% CO_2_. Cells infected with *C. muridarum* were grown for 72 h in the presence of 1 μg/mL of cycloheximide. Infected cell monolayers were detached by scraping and disrupted by sterile glass beads to lyse the host cells and release elementary bodies (EBs). Cell debris was removed by centrifugation at 500 × g for 15 min. *Chlamydia* EBs were purified in a sucrose urografin gradient [bottom layer 50% (w/v) sucrose solution; top layer, 30% (v/v) urografin in 30 mM Tris–HCl buffer, pH 7.4]; centrifugating at 9,000 × g and 25°C for 60 min. After one-wash step with 0.2 μm filtered PBS (pH 7.4), purified EBs were pelleted and resuspended in an isotonic sucrose-phosphate-glutamate (SPG) buffer and aliquots were stored frozen at −80°C. Infectious titers of this suspension were determined by titration on LLCMK2 cell monolayers. In brief, after 48 h of incubation, infected cells were fixed with methanol 100% and then air dried. Chlamydial inclusions were stained with FITC-monoclonal antibodies against chlamydial LPS (Biomerieux, Marcy l'Etoile, France) and analyzed by fluorescence microscopy. Chlamydial LPS (cLPS) was kindly supplied by Dr. Adrian Eley (UK). cLPS was extracted and further repurified as previously described (29, 30).

### Inoculation of Animals

Six to Eight week old male NOD, C57BL/6 (B6), BALB/c, μMT, IL-10^−/−^, TLR2^−/−^, and TLR4^−/−^ mice were purchased from Jackson Laboratory and then bred and maintained under specific pathogen free conditions in the vivarium of the CIBICI, Universidad Nacional de Cordoba, Argentina. μMT, IL-10^−/−^, TLR2^−/−^, and TLR4^−/−^ mice were on B6 background. Animals were maintained in a 16 h light, 8 h dark cycle, at 20 ± 2°C, with food and water *ad libitum*. All experiments were approved by and conducted in accordance with guidelines of the Committee for Animal Care and Use of the Facultad de Ciencias Químicas, Universidad Nacional de Córdoba (Res. HCD 240/216), in strict accordance with the recommendation of the Guide for the Care and Use of Laboratory Animals of the NIH (NIH publication 86–23). All efforts were made to minimize suffering and discomfort. As previously described (25), mice were inoculated in the meatus urethra with 1 × 10^8^ EBs of *C. muridarum* in 20 μL of SPG. For the inoculation, animals were anesthetized and put on their backs, the prepuce was pulled back and the inoculum was placed on the meatus urethra, simulating its natural infection path to the genital tract. A control group of animals was sham infected with 20 μL of SPG. This time point was considered day 0 of infection. Animals from the infected and control groups were euthanized at different days post-infection (dpi) according to the parameters under study.

### Detection of *Chlamydia* spp. by Polymerase Chain Reaction (PCR)

Qualitative and quantitative detection of *C. muridarum* was performed by conventional and real time PCR, respectively, by the amplification of the *C. muridarum omp1* gene (25, 28). Male genital tract tissues from infected and control animals were obtained at different times pi. Total DNA was extracted from tissue samples using commercially available extraction columns (AccuPrep® Genomic DNA Extraction Kit, Bioneer, Daejeon, Korea) following the specifications provided by the manufacturer. The primers used for amplification of a 100-bp fragment of the *omp1* gene were: sense 5′-GCC GTT TTG GGT TCT GCT T-3′ and antisense 5′-CGT CAA TCA TAA GGC TTG GTT CA-3′. The primers used for amplification of a 185 bp fragment of the *eef2* housekeeping gene were as follows: sense 5′-AAG CTG ATC GAG AAG CTG GA-3′ and antisense 5′-CCC CTC GTA TAG CAG CTC AC-3′. Conventional PCR was performed as previously described (25). A sample was considered positive when amplification of the *omp1* gene was detected. Quantitative PCR was performed on a StepOne™ instrument (Life technologies, Carlsbad, CA, USA) using SYBR® Select Master Mix (Life technologies). Each qPCR experiment was performed at least in triplicate, in a final volume of 15 μL. Each reaction contained 2 μL of DNA template and 1 μM of each primer (sense/antisense) of both genes (the *omp1* target gene and the *eef2* housekeeping gene). A single initial denaturation step of 15 min at 95°C was followed by 40 cycles of 15 s at 95°C (denaturation), 30 s at 60°C (annealing), and 30 s at 72°C (elongation). Negative and positive controls were included in each run. After performing thermal cycling, qPCR amplification data were analyzed using StepOne software (Applied Biosystems, Foster City, CA, USA).

### Spleen and Lymph Node Cell Cultures

Single mononuclear cell suspensions from spleen or prostate draining lymph nodes of individual mice were obtained by Ficoll-Paque PREMIUM 1.084 centrifugation gradients (GE Healthcare Bio-Sciences AB, Uppsala, Sweden). Live cells were counted by trypan blue exclusion, resuspended in RPMI 1640-GlutaMAX (Life Technologies), supplemented with 0.1% gentamicin (50 mg/mL), 50 mM 2-ME (Life Technologies), and 10% FBS and incubated in the presence of heat inactivated *C. muridarum* EBs for 48 h (multiplicity of infection, MOI 1000) or medium alone. All cell combinations were cultured at density of 0.5 × 10^6^ cell/ml per well and set up in triplicate. Plates were incubated for 48 h at 37°C in a water saturated 5% CO_2_ atmosphere. After the incubations, cytokine concentration or frequency of cytokine-producing cells were assayed using ELISA kits or by flow cytometry, respectively.

### Flow Cytometry

For cell surface staining, 10^6^ cells were labeled with 50–100 μl of a mix of mAbs at proper concentrations in PBS containing 3% FCS for 20–30 min at 6–8°C in the dark. The following antibodies were used: CD45, CD19, CD21, CD23, CD43, CD3, CD11b, CD11c, pErk, pStat3, PD1, PD-L1, CD39, FcγRIIb, and TLR4 (Biolegend or eBioscience, San Diego, CA, USA). Staining with the Fixable Viability Dye eFluor 780 (eBioscience) was performed before cell surface staining, according to the manufacturer's instructions. Intracellular IL-10 expression was visualized by immunofluorescence staining and analyzed by flow cytometry, as described (31). Briefly, isolated leukocytes or purified cells were resuspended (2 × 10^6^ cells/ml) in complete medium (RPMI 1640 media containing 10% FCS, 200 200 μ/ml penicillin, 200 U/ml streptomycin, 4 mM L-glutamine, and 0.05 mM 2-ME; all from Life Technologies) with LPS (10 μg/ml, *Escherichia coli* serotype 0111: B4; Sigma-Aldrich), PMA (50 ng/ml; Sigma-Aldrich), ionomycin (500 ng/ml; Sigma-Aldrich), and monensin (2 μM; eBioscience) for 5 h in 48-well flat-bottom plates. After cell staining for surface molecules, cells were fixed and permeabilized with CytofixCytoperm (BD Biosciences) and stained with anti-mouse IL-10 mAb (Biolegend) according to the manufacturer's instructions. Data were collected on LSR II or FACS-CANTO flow cytometers (BD Biosciences, San Jose, CA, USA) and analyzed using FlowJo software (TreeStar Inc., Ashland, OR, USA). Proper compensation using Fluorescence Minus One (FMO) controls were used.

### B Cell Purification and Culture

Single cell suspensions were obtained from spleen, stained with anti-mouse B220 and CD19 fluorochrome-conjugated Abs and then positively selected by CD19 and B220 expression using a FACSAria Cell Sorter (BD Biosciences). To purify different B subsets, sort gates were defined based on B220, CD19, CD21, CD23, and CD4 expression: Follicular B (FOB) cells (CD21^int^CD24^int^ CD23^+^), marginal zone B (MZB)-like cells (CD21^hi^CD24^hi^CD23^−^) and B1 cells (CD43^+^CD19^+^) (26, 32, 33). Sort purities were >97%. Following this procedure, purified B cells (CD19^+^ B220^+^) or B cell sub-populations (FOB, MZB-like and B1) were adjusted to a final concentration of 2 × 10^6^ cells/ml and cultured with heat inactivated EBs of *C. muridarum* for 48 h (multiplicity of infection, MOI 1000) or medium alone. The contents of IL-10 and total immunoglobulins in culture supernatants were measured by ELISA.

### Cytokine Quantification

Cytokine concentrations in culture supernatants were determined by ELISA using paired antibodies for murine IL-10, IL-6, TNFα, IL-17A, and IFNγ (eBiosciences) according to the manufacturer's instructions.

### Antibody Treatments

To neutralize IL-10 *in vivo*, mice were treated with an IgG1 antibody (clone JES5-2A5) as previously described (34). Mice were i.p. injected (500 μg/mouse) 1 day prior to *C. muridarum* inoculation and also on days 0, 2, and 4 pi.

To deplete B cells, mice were treated with the IgG2a anti-mouse CD20 Ab (clone 5D2, kindly provided by Genentech Inc., San Francisco, CA, USA) as previously described (35). The antibody was i.p. administered (50 μg/mouse, single dose) 10 days prior to *C. muridarum* inoculation. Depletion was confirmed by peripheral blood, spleen and lymph node CD19^+^ B cells staining and flow cytometry analysis. Depletion efficacy showed to be ≥90% in peripheral blood the day before inoculation.

To deplete MZB cells, mice were treated with the IgG1 anti-mouse Ly9.7.144 mAb as previously described (36). The antibody was i.p. administered (250 μg/mouse, single dose) 1 day before *C. muridarum* inoculation. Depletion was confirmed by spleen cells staining and flow cytometry analysis in infected and non-infected mice as previously described (36).

### Adoptive Transfer Experiments

Sorted splenic B cells (2 × 10^6^ cells in total) from WT or IL-10 ^−/−^ mice were i.v. injected into IL-10 ^−/−^ mice 1 day before *C. muridarum* inoculation. Mice were euthanized 7 days later. Chlamydial DNA was analyzed in prostate tissue as previously described (25).

### Statistical Analysis

Statistical analysis was performed using one-way ANOVA with Bonferroni *post hoc* test analysis. Data are shown as mean ± SEM in the graphs. Statistical tests were performed using the GraphPad Prism 5.0 software (GraphPad Software Inc., La Jolla, CA, USA). *P-*values ^*^ < 0.05, ^**^ < 0.01, and ^***^ < 0.001 were considered significant in all analyses.

## Results

### *C. muridarum* Induces the Secretion of IL-10 by Mononuclear Cells Early After Infection

**Figure 1:**
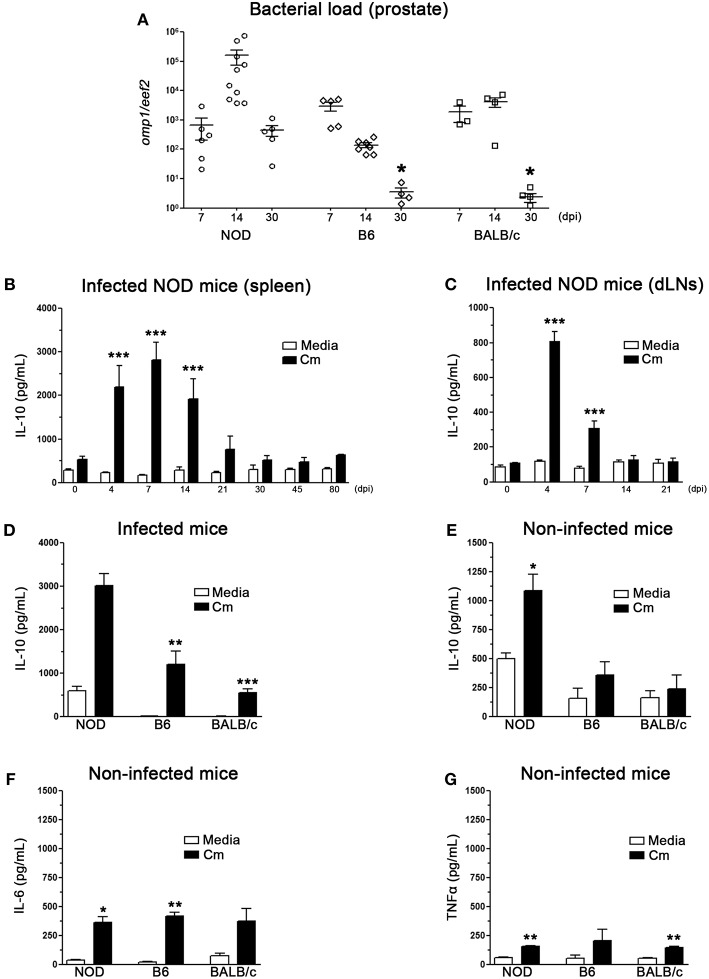
IL-10 production during C. muridarum infection of the male genital tract. **(A)** Quantitative PCR determination of chlamydial DNA (*omp1* gene) in prostate tissue from infected NOD, B6, and BALB/c mice. Mice were inoculated with *C. muridarum* and euthanized at different time points after infection as indicated in the figure. Quantitative determination of chlamydial DNA (*omp1* gene) was performed by real time PCR and the results are depicted as the relative expression to the *eef2* housekeeping gene. **(B–E)** IL-10 levels measured by ELISA in culture supernatants of spleen **(B)** or prostate draining lymph node **(C)** cells from *C. muridarum* infected NOD mice at different dpi. IL-10 was measured after *in vitro* stimulation with heat killed *C. muridarum* (black bars) or media (white bars). IL-10 in culture supernatants of spleen cells from infected **(D)** or non-infected **(E)** NOD, B6, and BALB/c mice after heat killed *C. muridarum* stimulation (black bars) or media (white bars). IL-6 **(F)** and TNFα **(G)** measured by ELISA in culture supernatants from spleen mononuclear cells from non-infected NOD, B6, and BALB/c mice. Spleen and prostate draining lymph node cells were *in vitro* cultured for 48 h with medium alone (media) or in presence of heat killed *C. muridarum* (Cm). Data are shown as mean ± SEM, *n* ≥ 5 per group, and representative of 3 independent experiments with essentially the same results. Statistical analysis was performed using one-way ANOVA with Bonferroni *post hoc* test analysis as appropriate. ^*^p < 0.05, ^**^p < 0.01 and ^***^p < 0.001.

Although there are data about different susceptibility in *Chlamydia* female models of infection using strains such as B6, BALB/c and others, there are almost no studies that analyze male infection in different strains of mice. Previously, we described that *C. muridarum* causes an ascending infection of the male genital tract preferentially colonizing the prostate gland (25). To analyze male genital infection and its relation with IL-10 production in different strains of mice we first analyzed bacterial load in prostate tissue from infected mice of B6, BALB/c, and NOD mice on different dpi. As shown in Figure 1A, B6 and BALB/c mice showed significantly lower bacterial loads than NOD mice at 30 dpi, indicating that they cleared the infection more quickly and efficiently than NOD mice.

The kinetics of IL-10 production was also analyzed at different time-points after male genital tract infection with *C. muridarum*. Spleen and prostate draining lymph node cells (dLNs) from infected and non-infected animals were *in vitro* stimulated with heat inactivated *C. muridarum* as previously reported (25) and IL-10 concentration in culture supernatants was analyzed by ELISA (Figures 1B–E). High levels of IL-10 were detected in culture supernatants of mononuclear cells (either from spleen or prostate draining lymph nodes) from infected NOD at early days after infection, reaching peak levels at 4–7 dpi (Figures 1B,C). Notably, splenic mononuclear cells produced much more IL-10 than prostate draining lymph node cells. Spleen mononuclear cells from infected B6 and BALB/c mice also secreted IL-10 when *in vitro* stimulated with heat inactivated *C. muridarum*. However, these values were significantly lower than those observed in cell culture supernatants from NOD mice (Figure 1D). Although increased secretion levels of IL-10 were also observed when spleen mononuclear cells from non-infected NOD mice were stimulated with heat inactivated *C. muridarum*, these levels were much lower than those found when assaying spleen cells from infected mice (compare values between Figures 1E,D). Moreover, no significant differences in IL-10 levels were detected in culture supernatants of spleen cells from non-infected B6 and BALB/c mice (Figure 1E). Also, increased levels of IL-6 and TNFα secretion were detected in culture supernatants of spleen cells from non-infected mice of all strains after *in vitro* stimulation with heat inactivated *C. muridarum*, with no differences between strains (Figures 1F,G).

Altogether, these results indicate that *C. muridarum* induces IL-10, IL-6 and TNFα production by mononuclear cells. Moreover, those mice that showed the highest capability to secrete IL-10 (NOD mice) were the least efficient in clearing the infection.

### B Cells Are the Main Source of IL-10 After *C. muridarum* Stimulation

**Figure 2:**
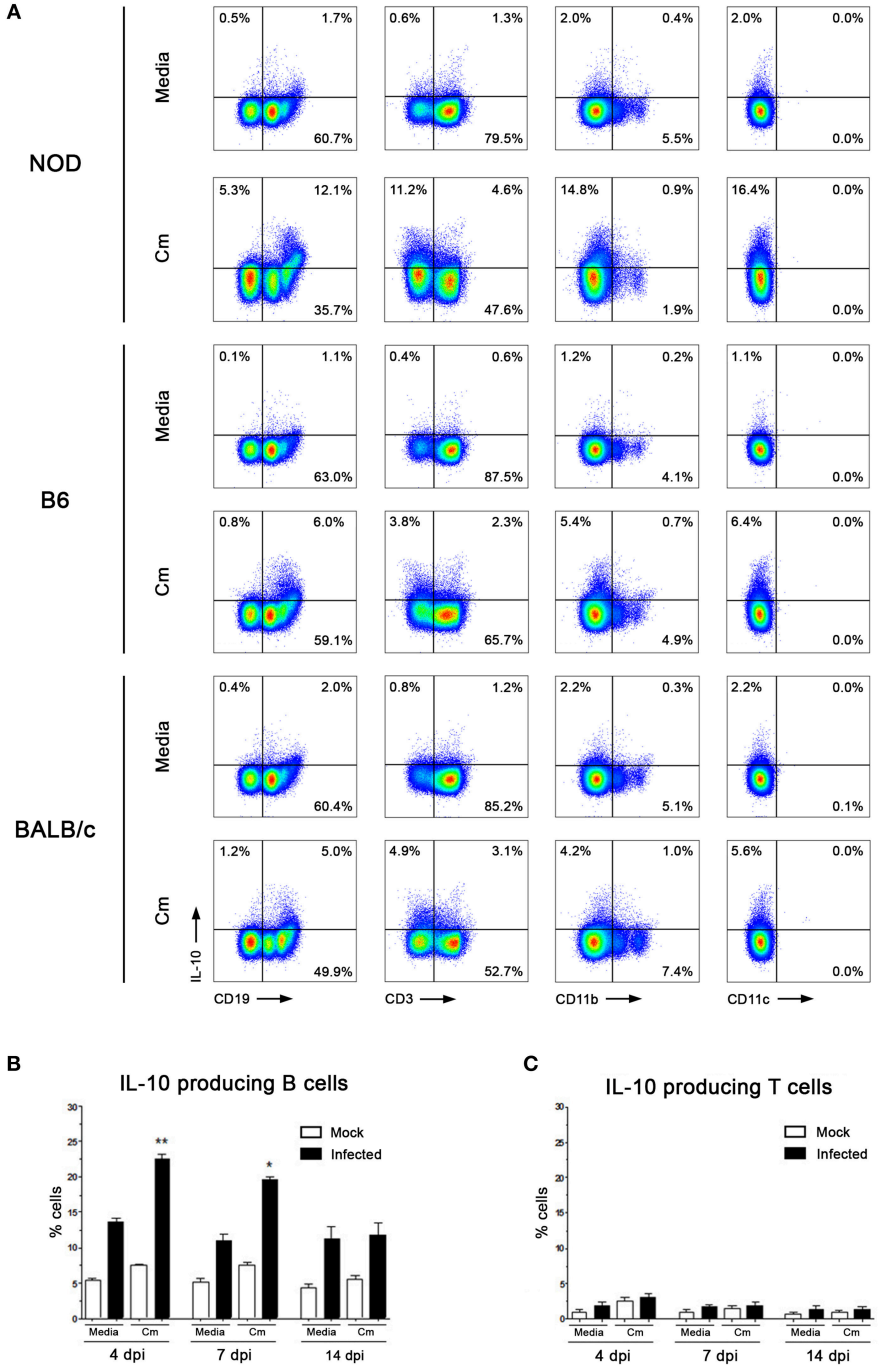
B cells are the main source of IL-10 secretion after *C. muridarum* stimulation. **(A)** Percentage of IL-10 producing cells among CD19^+^, CD3^+^, CD11b^+^, and CD11c^+^ cells. Spleen mononuclear cells from non-infected NOD, B6, and BALB/c mice were *in vitro* stimulated with media or heat killed *C. muridarum* (Cm) for 48 h and IL-10^+^ cells were analyzed by flow cytometry. Since all IL-10 producing cells were CD45^+^, the analysis was performed gating CD45^+^ cells. Intracellular IL-10 expression was visualized by immunofluorescence staining after a short incubation with PMA and inomycin as previously described (31). Proper compensation using Fluorescence Minus One (FMO) controls were performed. Only viable cells were analyzed by the use of Fixable Viability Dye eFluor 780. **(B)** IL-10^+^CD19^+^ and **(C)** IL-10^+^CD3^+^ cells from spleen of non-infected (mock) or infected NOD mice analyzed at different dpi. Spleen mononuclear cells were *in vitro* stimulated with heat killed *C. muridarum*. Data are shown as mean ± SEM, *n* ≥ 5 per group, and representative of 3 independent experiments with essentially the same results. Statistical analysis was performed using one-way ANOVA with Bonferroni *post hoc* test analysis as appropriate. ^*^p < 0.05, ^**^p < 0.01.

To identify which immune cells produced IL-10 in response to *C. muridarum*, we analyzed the phenotypic characteristics of IL-10 producing cells both *in vitro* and *in vivo*. Our first strategy was to *in vitro* stimulate spleen and dLNs mononuclear cells from non-infected NOD, B6 and BALB/c mice and then perform Abs staining for FACS analysis. Because preliminary experiments yielded similar results in spleen and dLNs, and also due to low cell numbers in dLN samples, we performed most of the experiments with spleen mononuclear cells. Since all IL-10 producing cells were CD45^+^ (data not shown), we analyzed the expression of IL-10 and CD19, CD3, CD11b, and CD11c within CD45^+^ cells (Figure 2A). Mononuclear cells stimulated with *C. muridarum* showed a higher frequency of IL-10 producing cells than unstimulated cells (media). IL-10 producing cells were mostly CD19^+^ cells although less frequencies of CD3^+^ or CD11b^+^ cells were also detected (Figure 2A). On the contrary, no IL-10 producing CD11c^+^ cells were observed (Figure 2A). When comparing mice strains, higher frequencies of IL-10 producing cells were detected in *C. muridarum* stimulated cells from NOD mice with respect to B6 and BALB/c mice (Figure 2A). Remarkably, CD19^+^ cells were the major producers of IL-10 in every mice strain analyzed. Taking into account that these data come from experiments assaying mononuclear cells from non-infected mice, results suggest that some *C. muridarum* component is *per se* able to trigger IL-10 production, even if B cells have not previously contacted chlamydial antigens.

We next monitored IL-10^+^CD19^+^ and IL-10^+^CD3^+^ cells at different time points after infection. In concordance with results described in Figure 1A, higher percentages of IL-10^+^CD19^+^ cells from spleen were detected in infected NOD mice during the first days of the experimental schedule (Figure 2B). Moreover, no significant changes in the IL-10^+^CD3^+^cell subset were observed (Figure 2C).

**Figure 3:**
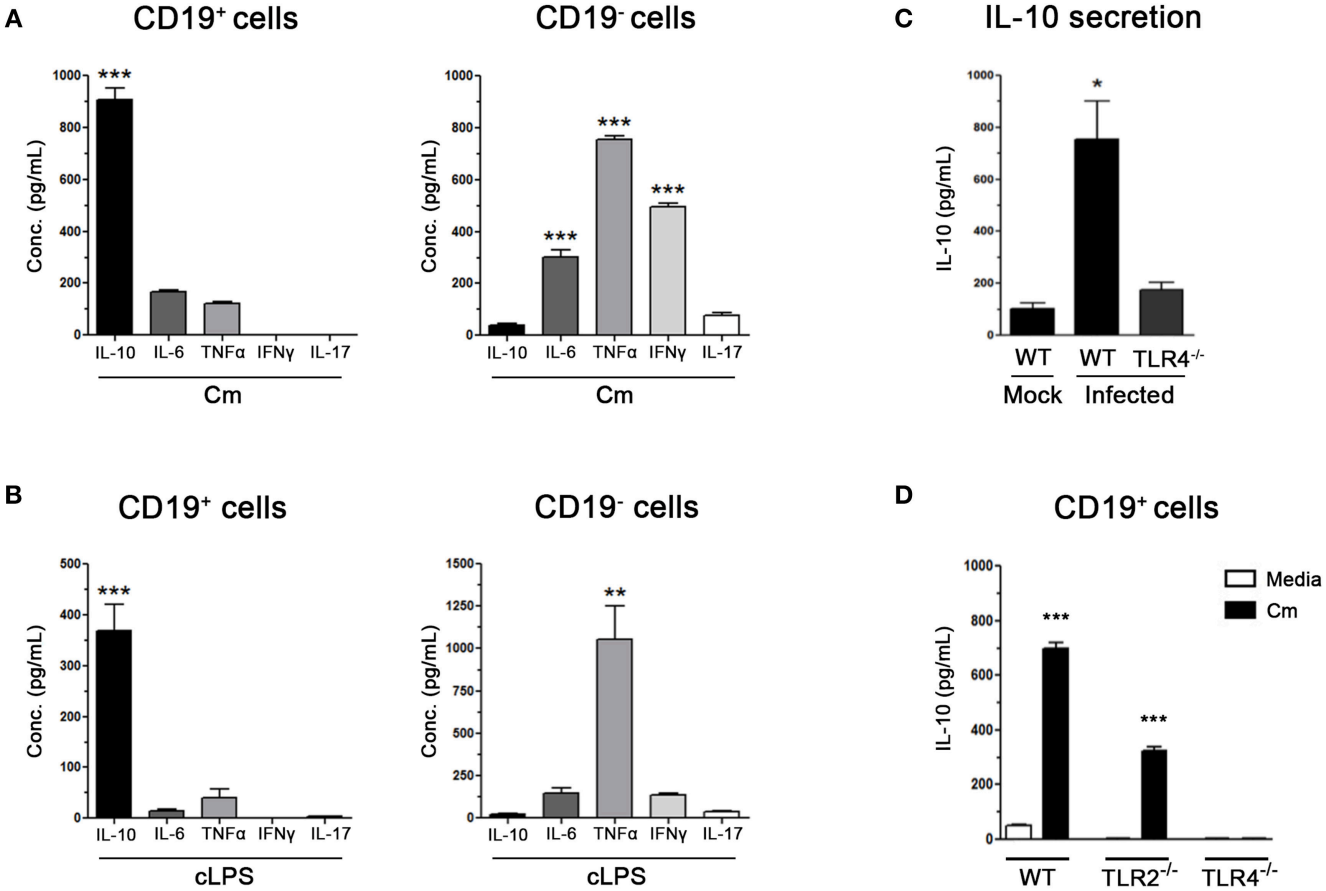
Purified B cells produce IL-10 through the engagement of innate immune receptors after *C. muridarum* stimulation. **(A)** Cytokine levels measured by ELISA in culture supernatants from sorted purified spleen CD19^+^ or CD19^−^ cells (purity >97%) after *in vitro* stimulation with *C. muridarum* **(B)** Cytokine levels measured by ELISA in culture supernatants from purified spleen CD19^+^ or CD19^−^ cells (purity >97%) after *in vitro* stimulation with chlamydial LPS (cLPS) **(C)** IL-10 levels measured by ELISA in culture supernatants of spleen mononuclear cells from *C. muridarum*-infected TLR4^−/−^ and wild type (B6) mice at 4 dpi. **(D)** IL-10 production by spleen sorted CD19^+^ purified B cells from wild type (B6), TLR2^−/−^ and TLR4^−/−^ mice after *in vitro* stimulation with media or heat killed *C. muridarum*. Data are shown as mean ± SEM, *n* ≥ 5 per group, and representative of 3 independent experiments with essentially the same results. Statistical analysis was performed using one-way ANOVA with Bonferroni *post hoc* test analysis as appropriate. ^*^p < 0.05, ^**^p < 0.01 and ^***^p < 0.001.

To verify the results described above, different experiments were performed. On the one hand, splenic CD19^+^ (purity >97%) and CD19^−^ cells were sorted from non-infected NOD mice and then *in vitro* stimulated with heat inactivated *C. muridarum*. High levels of IL-10, accompanied by slightly detectable amounts of IL-6 and TNF-α, were detected in culture supernatants from stimulated CD19^+^ cells (Figure 3A). On the contrary, minimum IL-10 production and significantly higher IL-6, TNF-α and INFγ levels were detected in culture supernatants from *C. muridarum* stimulated CD19^−^ cells (Figure 3A). As IL-10 production by B cells could be triggered through Toll-like receptor engagement (36), we assessed IL-10 secretion after chlamydial LPS (cLPS) stimulation of CD19^+^and CD19^−^ cells. High contents of IL-10 were detected in culture supernatants from CD19^+^ cells, while only high levels of TNF-α were detected in culture supernatants from cLPS stimulated CD19^−^ cells (Figure 3B).

On the other hand, and to confirm the participation of TLR-4 in this process, the secretion of IL-10 by spleen mononuclear cells of infected TLR4^−/−^ and wild type (B6) mice was *ex vivo* analyzed after *C. muridarum* stimulation. Markedly high levels of IL-10 were secreted by mononuclear cells from infected wild type mice (Figure 3C); however, this effect was completely abrogated when cells lacked TLR4 expression, showing similar secretion levels to those observed in mononuclear cells from mock infected wild type mice (Figure 3C). In agreement, sorted CD19^+^ cells from TLR4^−/−^ mice exhibited a complete inability to secrete IL-10 when they were *in vitro* stimulated with *C. muridarum* (Figure 3D), indicating that B cells need TLR4 to produce IL-10 in response to *C. muridarum*. However, it should not be ruled out the possible participation of TLR4 in the production of other cytokines in addition to IL-10. Moreover, TLR2 signaling showed to be partially involved since purified B cells from TLR2^−/−^ mice produced significantly higher amounts of IL-10 amounts when stimulated with *C. muridarum*, although these levels were much lower than those from cells from wild type mice (Figure 3D). Altogether, these data indicate that B cells are the major source of IL-10 and that bacterial components trigger its secretion through the engagement of innate immune receptors.

### MZB Cells Are the Main IL-10 Producers

**Figure 4:**
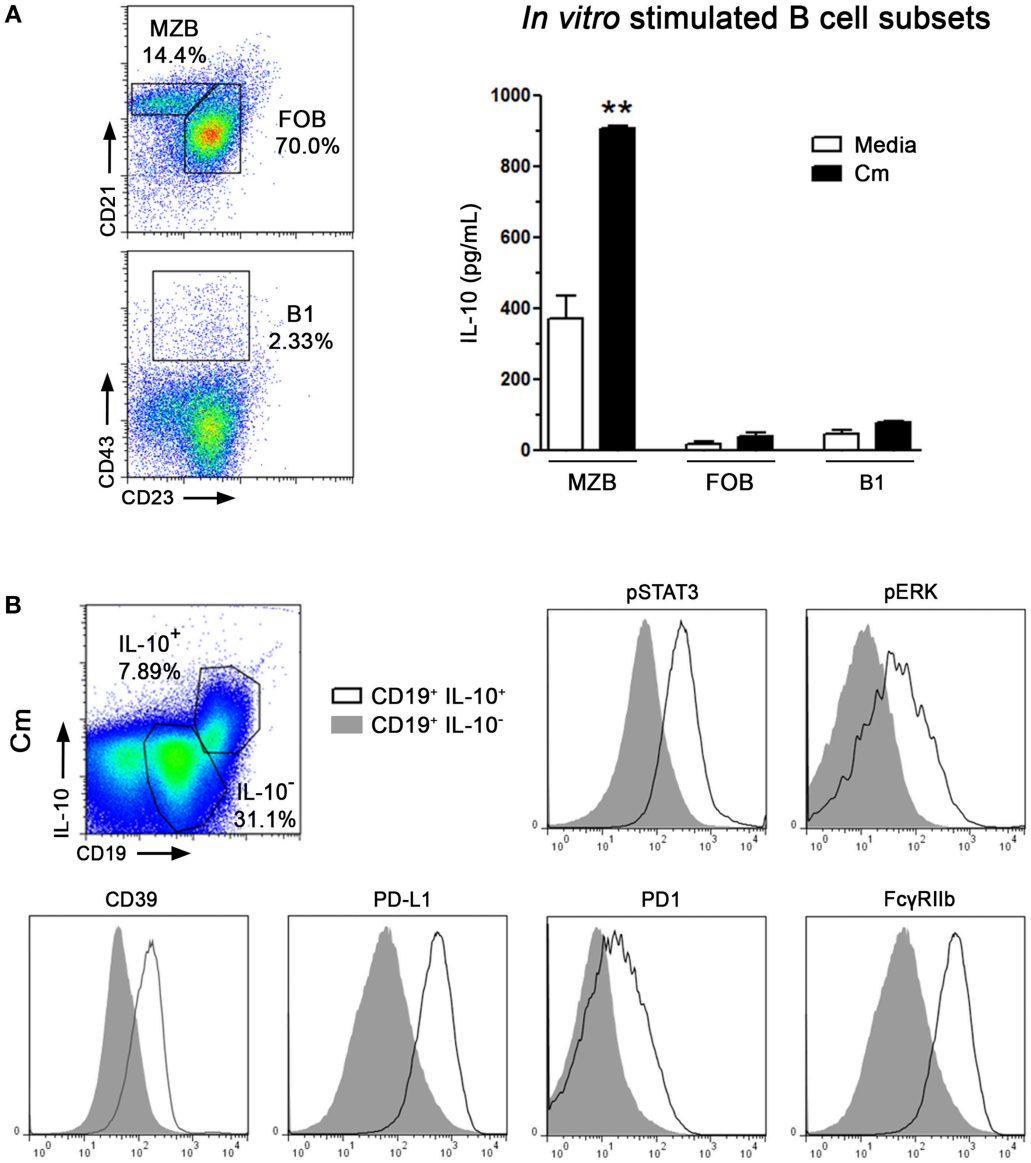
Marginal Zone B lymphocytes are the main IL-10 producers after C. muridarum stimulation. Spleen MZB, FOB, and B1 cell subsets were sorted according to the expression of characteristic surface markers. **(A)** Representative staining of Marginal Zone (CD21^hi^, CD23^−^, CD19^+^), Follicular (CD21^int^, CD23^+^, CD19^+^), and B1 (CD43^+^CD23^+^ CD19^+^) cell subsets from gated CD19^+^ B lymphocytes. Purified B subsets were *in vitro* cultured in presence of heat killed *C. muridarum* (black bars) or media alone (white bars) for 48 h and IL-10 contents were measured by ELISA. **(B)** Representative gates from IL-10^+^CD19^+^ or IL-10^−^CD19^+^ cells from NOD spleen mononuclear cells after *in vitro* stimulated with heat killed *C. muridarum*. Histograms showing the expression of pSTAT3, pERK, CD39, PD-L1, PD1, and FcγRIIb in IL-10^+^ and IL-10^−^gated B cells. Data are shown as mean ± SEM, *n* ≥ 5 per group, and representative of 2 independent experiments with essentially the same results. Statistical analysis was performed using one-way ANOVA with Bonferroni *post hoc* test analysis as appropriate. ^**^p < 0.01.

In mice, three mature splenic B cell subsets with different functional activities, phenotypes and/or topographic locations have been identified so far: B1, FOB and MZB cells (37, 38). Thus, as indicated in Figure 4A, spleen B cell subsets were sorted according to the expression of characteristic surface markers and then *in vitro* stimulated with *C. muridarum* as described above. As shown in Figure 4A, high concentration of IL-10 was detected in culture supernatants of MZB-like cells, whereas no significant IL-10 amounts were detected in culture supernatants of either FOB or B1 cells. Phenotype of IL-10 B subsets from dLNs was not evaluated. To further analyze the phenotypic characteristics of IL-10 producing cells we searched for the expression of different molecules after *C. muridarum* stimulation and found higher pSTAT3 and pERK immunostaining together with enhanced surface expression of CD39, PD-L1, PD1 and FcγRIIb in CD19^+^IL-10^+^ cells when compared to CD19^+^IL-10^−^ cells (Figure 4B). Altogether, these data indicate that *in vitro* MZB-like cells are the major IL-10 contributors after *C. muridarum* stimulation. Moreover, IL10-secreting B cells express different molecules involved in immune regulation.

### IL-10 Producing B Cells Delay Bacterial Clearance

**Figure 5:**
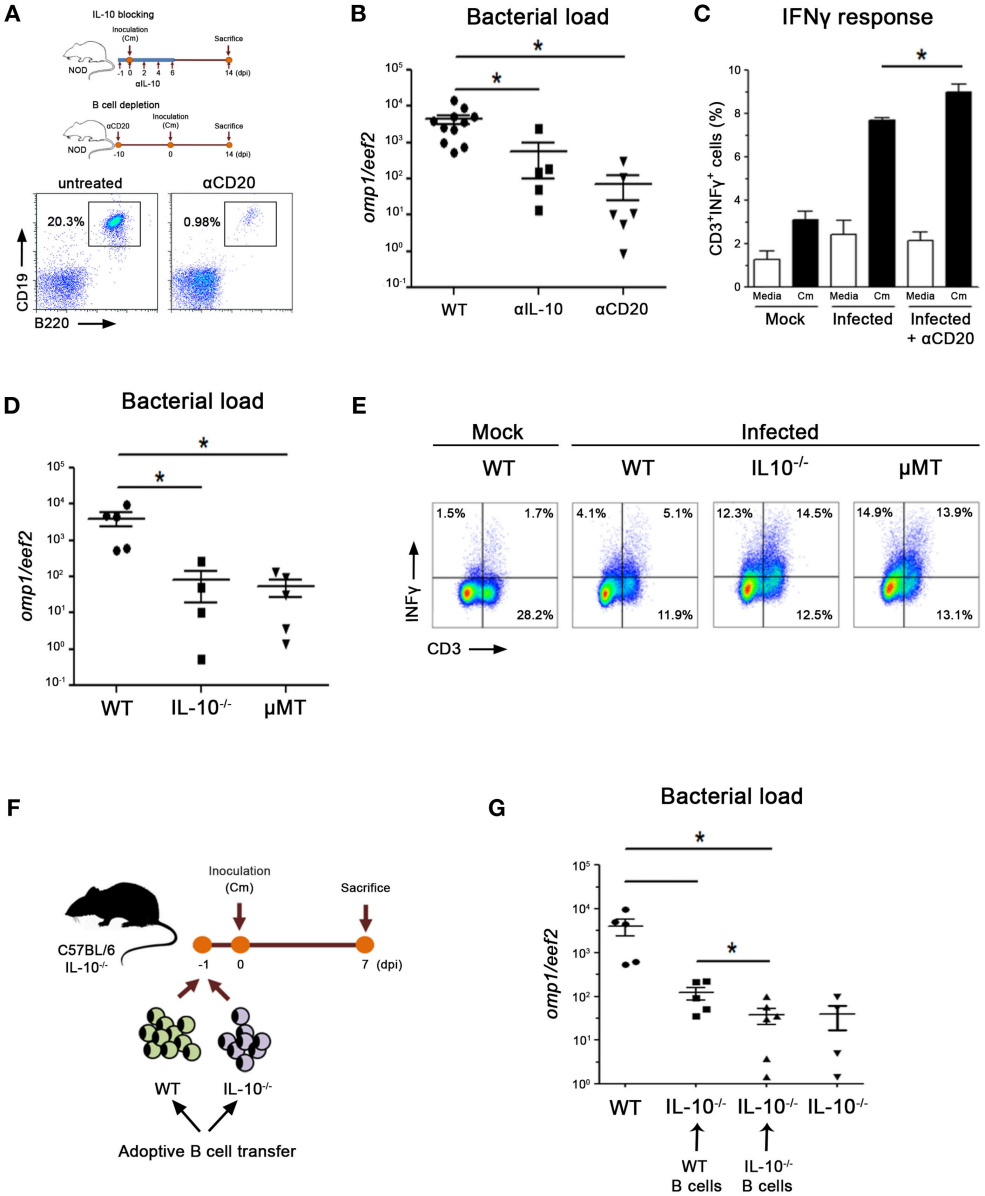
IL-10-producing B cells contribute to delay bacterial clearance. **(A)** Experimental scheme showing mice treatments with neutralizing αIL-10 or depleting αCD20 antibodies. Representative dot plots showing CD19^+^B220^+^ cell frequencies in spleen from untreated and αCD20 treated mice show depletion efficiency >90% at day 0. Mice were inoculated with *C. muridarum* at 0 dpi and euthanized at 14 dpi. **(B)** Prostate bacterial load assessed by quantitative PCR determination of chlamydial DNA (*omp1* gene) in prostate tissue from αIL-10-, αCD20-treated or untreated wild type NOD mice. **(C)** Percentage of IFNγ-producing CD3 cells in spleen cultures from non-infected (mock), infected or infected and treated NOD mice after *in vitro* stimulation with media (white bars) or heat inactivated *C. muridarum* (Cm, black bars). **(D)** Prostate bacterial load assessed by quantitative PCR determination of chlamydial DNA (*omp1* gene) in prostate tissue from infected wild type (B6) mice, mice deficient in IL-10 (IL-10^−/−^) or deficient in B cells (μMT) euthanized at day 7 dpi. **(E)** Representative dot plots showing CD3^+^IFNγ^+^ cell frequencies in spleen mononuclear cell cultures from non-infected (mock) or infected mice after *in-vitro* stimulation with heat killed *C. muridarum* (gated CD3 cells). **(F)** Experimental scheme of adoptive B cell transfer into IL-10^−/−^ mice. **(G)** Prostate bacterial load assessed by quantitative PCR determination of chlamydial DNA (*omp1* gene) in prostate tissue from infected IL-10^−/−^ mice that received wild type or IL-10-deficient B cells. Data are shown as mean ± SEM, *n* ≥ 5 per group, and representative of 3 independent experiments with essentially the same results. Statistical analysis was performed using one-way ANOVA with Bonferroni post hoc test analysis as appropriate. ^*^p < 0.05.

To gain understanding of the possible IL-10 and B cell contribution to bacterial clearance in NOD mice, we performed IL-10 blocking or B cell depletion *in vivo* experiments by administering mice with anti-IL10-neutralizing or anti-CD20-depleting antibodies, respectively (Figure 5A). B cell depletion efficacy showed to be ≥90% in spleen from NOD mice (Figure 5A). He administration of IL-10 blocking antibodies during the first days of the experimental scheme resulted in significantly lower prostate bacterial loads when compared with untreated mice (Figure 5B). In addition, the anti-CD20 Ab treatment resulted in even lower bacterial loads indicating that in the presence of low bioavailable IL-10 or low B cell counts during the first days of the experimental scheme, infected animals can clear bacteria from prostate tissue more efficiently (Figure 5B). To assess if this improvement in clearing the infection was associated to the induction of enhanced Th1 immune responses, we analyzed the levels of INFγ producing T cells. Indeed, elevated frequencies of INFγ^+^ CD3^?^ cells were detected in spleen from αCD20 Ab treated mice (Figure 5C). Thus, anti CD20 Ab treatment conferred NOD mice the capability of inducing more robust Th1 responses and clearing the infection more efficiently.

A preferential *C. muridarum* colonization of the prostate is also observed in B6 mice (25). In concordance with bacterial loads observed in αIL10 or αCD20 treated NOD mice, B6 mice deficient in IL10 (IL10^−/−^) or B cells (μMT) exhibited lower bacterial loads in prostate tissue than their wild type (B6) counterparts (Figure 5D). In agreement, both IL-10^−/−^ and μMT mice showed higher frequencies of INFγ-producing T cells in the spleen (Figure 5E). Finally, we performed adoptive transfer experiments in which purified B cells from WT or IL10^−/−^ mice were transferred into IL-10^−/−^ recipients (Figure 5F). When IL-10^−/−^ mice received B cells able to produce IL-10 (WT B cells), higher bacterial loads were detected in prostate tissue (Figure 5G). However, IL-10^−/−^ mice that received B cells unable to produce IL10 (IL-10^−/−^ B cells) showed similarly reduced bacterial loads to those observed in infected IL-10^−/−^ mice (Figure 5G).

These data suggest that in the absence of B cells, bioactive IL-10 or IL-10 producing B cells, *C. muridarum* clearance is achieved more efficiently, a process concomitant to the generation of robust Th1 immune responses.

### MZB Cells Impair Bacterial Clearance Ability

**Figure 6:**
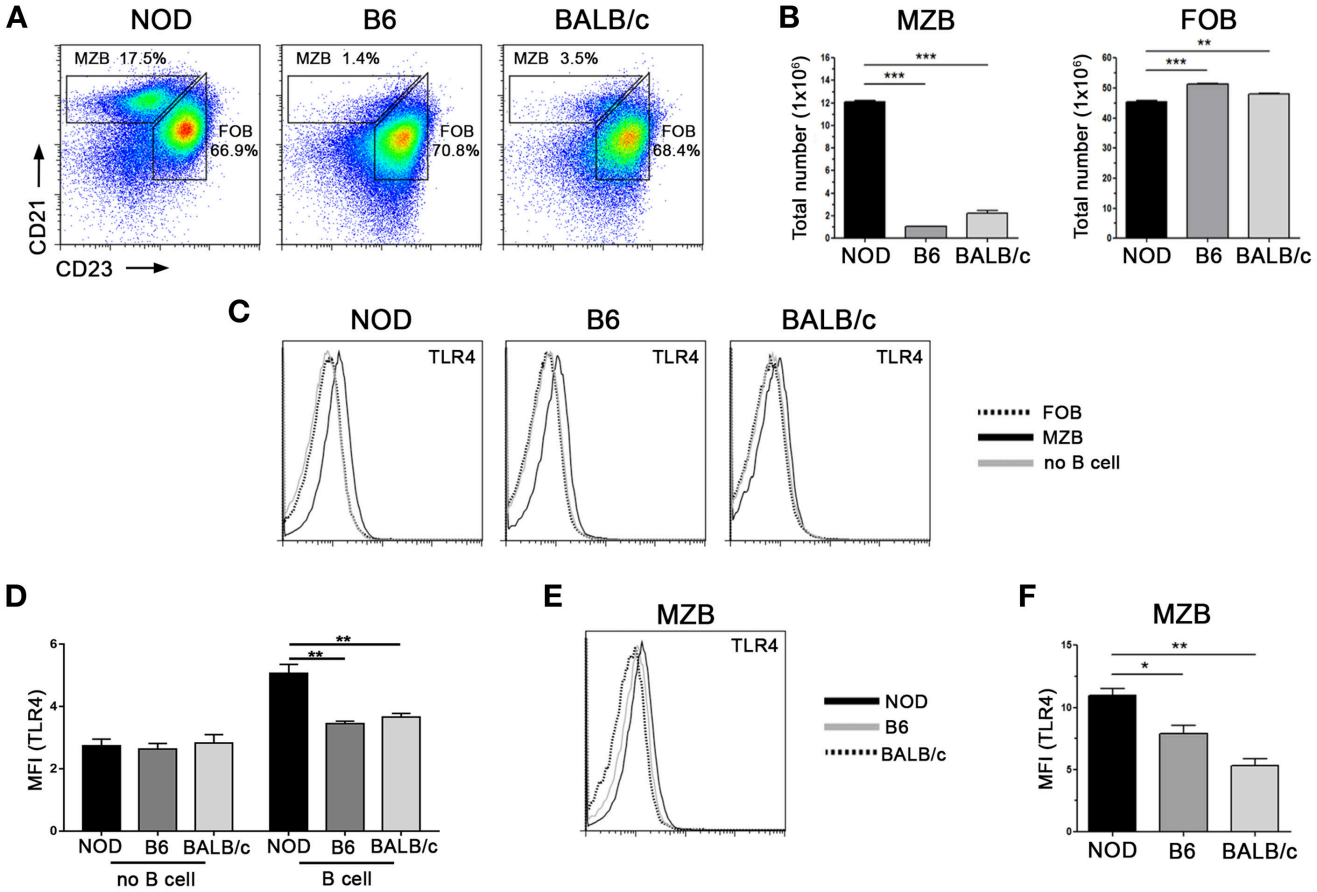
NOD mice have a higher frequency of MZB cells and also enhanced TLR4 expression. **(A)** Representative staining of Marginal Zone (CD21^hi^, CD23^−^) and Follicular (CD21^low^, CD23^+^) cell subsets from gated CD19^+^ B lymphocytes from NOD, B6 and BALB/c mice. **(B)** Total numbers of MZB and FOB cells in spleen from NOD, B6 and BALB/c mice. **(C)** Representative histograms showing TLR4 expression on splenic FOB, MZB, and non-B cells from NOD, B6, and BALB/c mice. **(D)** TLR4 mean fluorescence intensity (MFI) on B cells and no B cells from NOD, B6 and BALB/c mice **(E)** Representative histograms showing TLR4 expression on MZB cells from NOD, B6 and BALB/c mice. **(F)** TLR4 mean fluorescence intensity (MFI) on MZB cells from NOD, B6, and BALB/c mice. Data are shown as mean ± SEM, *n* ≥ 5 per group, and representative of two independent experiments with essentially the same results. Statistical analysis was performed using one-way ANOVA with Bonferroni *post hoc* test analysis as appropriate. ^*^p < 0.05, ^**^p < 0.01 and ^***^p < 0.001.

Previously, we observed a higher frequency of IL-10 producing cells within MZB-like cell subpopulation (Figure 3A) and also that NOD mice showed the highest frequency of IL-10^+^ cells during the course of the infection (Figures 1B–D, 4A). We then aimed to quantify the frequencies of MZB-like and FOB cells in the spleen from NOD, B6, and BALB/c mice. As shown in Figure 6A, NOD mice showed significantly much more MZB-like cell frequencies and counts than the other mice strains. On the contrary, slight differences in FOB cell frequencies and counts were found (Figures 6A,B). Since results shown above suggested that IL-10 secretion by B cells was triggered through the engagement of innate receptors by bacterial PAMPs, we analyzed TLR4 expression in spleen B cells and no B cells and showed higher TLR4 expression only in B cells from NOD mice, while no differences were observed in no B cells between the strains under study (Figures 6C,D). We also analyzed TLR4 expression in B cell subsets. Enhanced TLR4 expression was observed in CD21^hi^CD23^−^ MZB-like cells when compared with CD21^int^CD23^+^ FOB cells in all mice strains under study (Figure 6C). Besides having a higher frequency of CD21^hi^CD23^−^ MZB-like cells, the MZB-like cells from NOD mice exhibited an increased TLR4 expression than MZB-like cells from B6 or BALB/c mice (Figures 6E,F).

**Figure 7:**
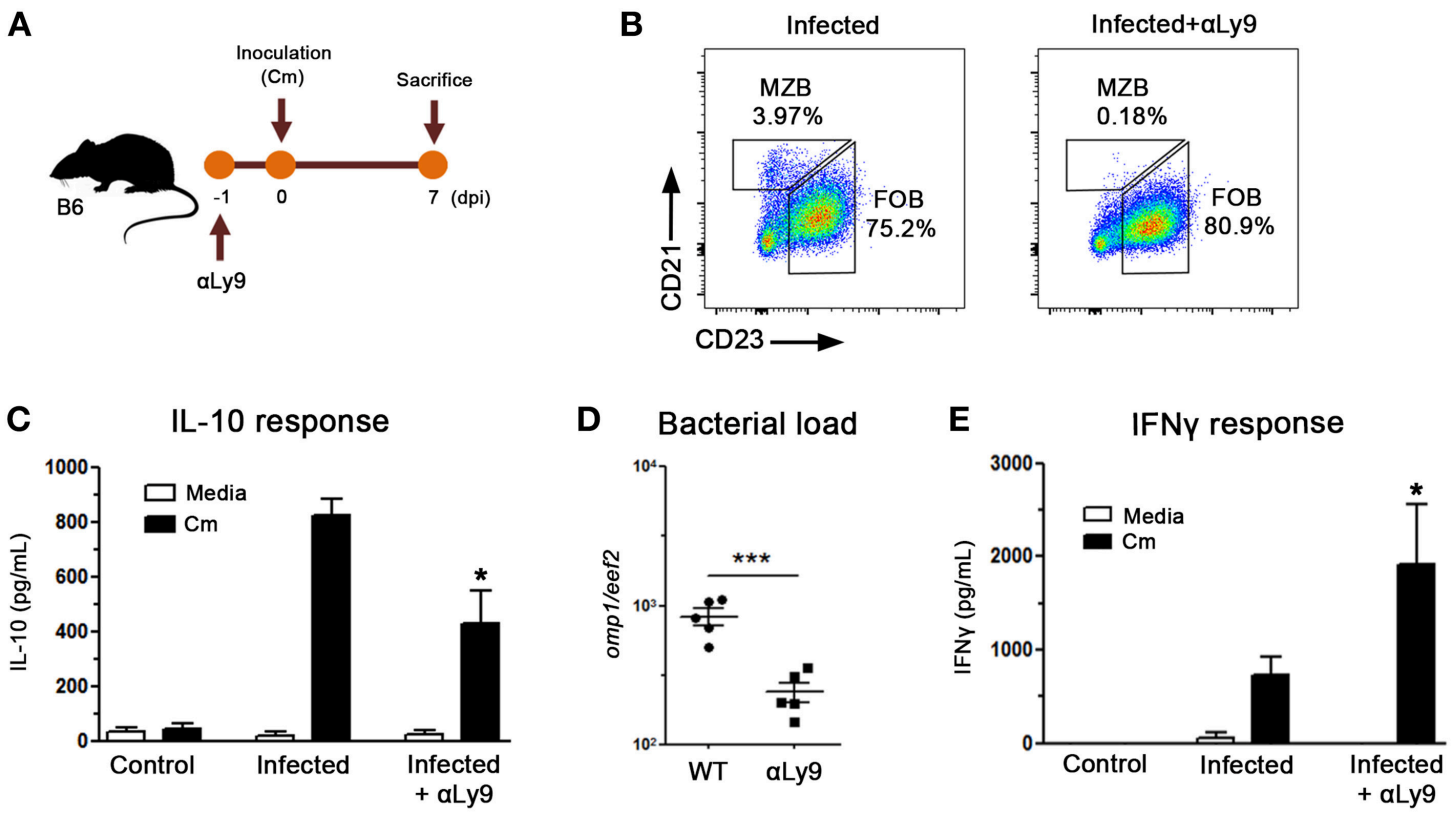
Marginal Zone B lymphocytes contribute to delay bacterial clearance. **(A)** Experimental scheme showing mice treatments with αLy9 antibody in B6 mice. Mice were inoculated with *C. muridarum* at 0 dpi and euthanized at 7 dpi. **(B)** Representative dot plots showing MZB cell depletion in αLy9-treated mice. **(C)** IL-10 levels measured by ELISA in culture supernatants of spleen mononuclear cells from *C. muridarum*-infected and αLy9-treated or untreated B6 mice at 4 dpi. Spleen mononuclear cells were *in vitro* cultured for 48 h with medium alone (media) or in presence of heat killed *C. muridarum* (Cm). **(D)** Prostate bacterial load assessed by quantitative PCR determination of chlamydial DNA (*omp1* gene) in prostate tissue from αLy9-treated or untreated B6 mice. **(E)** IFNγ levels in culture supernatants of spleen mononuclear cells from uninfected (control), infected or infected and αLy9-treated B6 mice after *in vitro* stimulation for 48 h with media (white bars) or heat killed *C. muridarum* (Cm, black bars). Data are shown as mean ± SEM, *n* ≥ 5 per group, and representative of two independent experiments with essentially the same results. Statistical analysis was performed using one-way ANOVA with Bonferroni *post hoc* test analysis as appropriate. ^*^p < 0.05, ^***^p < 0.001.

To assess the possible MZB cell implication in the delayed bacterial clearance observed in NOD mice, we performed *in vivo* experiments in which mice were treated with an anti-Ly9 antibody (Figure 7A), which has been reported to selectively eliminate MZB cells (35). As can be seen in Figure 7B, anti-Ly9 treated mice showed a significant reduction in MZB cells whereas minor changes were detected in the FOB compartment. When IL-10 production was analyzed in culture supernatants of spleen mononuclear cells from anti-Ly9 treated mice, significantly diminished IL-10 levels were observed, suggesting that MZB cells were important IL-10 contributors also *in vivo* (Figure 7C). Interestingly, the administration of anti-Ly9 antibodies resulted in significantly lower prostate bacterial loads and enhanced IFNγ production when compared to untreated mice (Figures 7D,E). These results suggest that the enhanced capacity to produce IL-10 exhibited by NOD mice is related to the presence of high MZB cell counts, thus postulating MZB cells as an important source of IL-10 secretion after *C. muridarum* stimulation.

## Discussion

IL-10 is a potent regulatory cytokine strongly implicated in the modulation of Th1 responses (39, 40). Evidence of a role for IL-10 in *Chlamydia* infections has been obtained from studies in patients and animal models (22–24, 26, 27, 41, 42). Certainly, increased IL-10 levels have been reported in endocervical secretions and semen from infected patients (39, 43, 44). Moreover, IL-10 gene polymorphisms associated with high IL-10 producing ability has been proposed as risk factor for persistent infection (45). Animal models have also provided compelling evidence of IL-10 production during *C. muridarum* infection of the female genital tract (26, 27, 46). Studies performed in mice deficient in IL-10, showed that the absence of this cytokine skews the anti-chlamydial immune response to a predominantly Th1 phenotype and prevented *Chlamydia*-induced immunopathology (22, 45, 47). Thus, cumulative data indicate that IL-10 has a critical role during *Chlamydia* infection (23). However, it is still unclear whether one type or multiple cell types actually provide that IL-10 rendering hosts less efficient in clearing the infection. In the present report, we provide compelling data indicating that an increased IL-10 production is observed early after the infection of the male genital tract with *C. muridarum*, being B cells the main source of that secretion. Although it cannot rule out that other regulatory cytokines could also be involved, our results demonstrate the regulatory participation of IL-10 in the early stages of the infection. The increased IL-10 production may skew the immune response away from a Th1 pattern thus impairing efficient bacterial clearance and favoring the establishment of chronic infections.

Evidence for a regulatory role of B cells was firstly described in a mouse model of inflammatory bowel disease (48). After that, several groups have reported that regulatory B cells play a role in diverse autoimmune, allergic and infectious conditions (49–53). The presence of IL-10 secreting B cells has been reported after *Schistosoma mansoni, Leishmania major, Brudia pahahgi, Listeria monocytogenes*, and other pathogen infection models in mice (53–56). In these studies, IL-10 secreting B cells were shown to mediate regulation of immune responses by inhibiting different immune cells such as neutrophils, NK cells, dendritic cells and inflammatory T cells (52–55). Also, IL-10 producing B cells have been reported in animal models of *C. muridarum* infection of the female genital tract (26). In detail, Moore-Connors et al. showed that after *Chlamydia* intravaginal infection, spleen CD43^+^IL-10^+^ or CD43^−^IL-10^+^ B cell subsets are induced (26). Authors focused their work on the characterization of both B cell populations and showed that CD43^+^ IL-10-producing B cells were induced via TLR signaling, while CD43^−^ IL-10-producing B cells required BAFF-mediated signals from dendritic cells for their differentiation and activation (26). Herein, using a different setting, we provided results that indicate the induction of IL-10-producing B cells after *Chlamydia* infection of the male genital tract. Our data show that B cells from NOD, B6 and BALB/c mice produce IL-10 upon *C muridarum* stimulation. Interestingly, male NOD mice, which exhibited the least efficient bacterial clearance ability with respect to other mice strains, were the highest IL-10 producers. More importantly, *in vivo* IL-10 neutralization or B cell depletion rendered NOD mice more efficient in clearing the infection suggesting a link between IL-10 secreting B cells and the establishment of chronic infections. The importance of IL-10 and B cells during *C muridarum* infection of the male genital tract was also indicated by the improved bacterial clearance ability observed in IL-10-deficient and μMT mice. However, caution should be taken when interpreting these data since IL-10 is absent not only in B cells in the first case, and defects in T cells, dendritic cells and innate immune system has been demonstrated in the second case (57). Nonetheless, our results strongly suggest that IL-10 producing B cells play an important role in the modulation of both the innate and adaptive immune response against *C muridarum*. Moreover, Su et al. also reported the induction of prominent IFNγ responses in *Chlamydia*-infected female μMT mice, although shedding of bacteria was not significantly different with respect to wild type mice (58). Our results also indicate that the TLR4 signaling pathway was essential for IL-10 production by B cells, in agreement with similar data reported in CD43^+^ B cells (26), where CD43^+^ IL-10-producing B cells displayed innate type features and were readily induced by *Chlamydia* via TLR signaling (26). In our work, findings showing IL-10 producing B cells from non-infected mice supports this data, since some *C. muridarum* component, such as cLPS, signaling via TLR4, could be triggering IL-10 production, even if B cells have not previously contacted chlamydial antigens. Moreover, evidence provided herein show that not only IL-10 but also PD1, PD-L1, CD39, and FcγRIIb expression are up-regulated on B cells after *Chlamydia* stimulation. Similarly, it has been reported that *Helicobacter felis*-stimulated B cells produce IL-10 and express the inhibitory PD1 molecule (59). This evidence strongly suggests that these inhibitory molecules could be also exerting immune regulation in our experimental model of infection, since they are well-known mediators of immune regulatory mechanisms (59, 60). Certainly, compelling evidence indicates that B regulatory cells regulate immunity not only through cytokine production but also via surface molecules such as CD39, CD73, and PD1 (40). In fact, a novel population of B cells has been shown to regulate colitis in an IL-10-independent manner but dependent on CD73 and CD39 (61).

Currently, available data indicate that there is no transcription factor or phenotypic cell marker specific for regulatory B cells (49). In mice, different subsets of IL-10-producing B cells have been reported, including CD5^+^CD1d^hi^ B cells, transitional 2 marginal-zone precursor (T2-MZP) cells, marginal zone B (MZB) cells, Tim^+^ B cells, CD138^+^ plasma cells and plasmablasts (49, 50, 62, 63). It has also been described that MZB cells rapidly respond to thymus-independent antigens, such as bacterial LPS (62). As CD5^+^ and MZB cells share many properties with innate immune cells, they have been named innate-like B cells. Interestingly, innate-like B cells produce high amounts of IgM and exhibit a rapid ability to produce high amounts of IL-10 following innate activation, being MyD88 and TLR signaling required for their regulatory activities (62).

In our experiments, IL-10-producing B cells induced after *C. muridarum* stimulation exhibited innate-like B cell characteristics since they were rapidly induced during the course of the infection and depended on TLR4 signaling. Noteworthy, our results show that NOD mice, which exhibited the highest frequencies of IL-10-secreting B cells, had the most abundant splenic MZB cell counts with enhanced TLR4 expression. In accordance, higher numbers of IL-10^+^ B cells have been described in NOD mice with respect to B6 mice (63, 64, 64). Moreover, treatment with anti-Ly9 antibody that deplete the MZB cell population rendered NOD mice more efficient in inducing enhanced Th1 immune responses and clearing the infection, thus supporting a regulatory role for this cell subset in our model of *Chlamydia* infection of the male genital tract. In agreement with our results, Moore-Connors et al. described two populations of IL-10 producing B cells during *C. muridarum* infection of the female genital tract, CD43^+^ and CD43^−^ IL10^+^B cells; the first one showing phenotypic characteristic of CD5^+^ and MZB cells (26). It could be speculated that those CD43^+^ cells, similar to the MZB cells in our settings, would be the relevant ones for the establishment of chlamydial chronic infections, since the anti-Ly9 antibody depleting therapy used in our experiments led to the induction of enhanced Th1 responses and improved bacterial clearance capability. However, the putative contribution of other IL-10 producing cells from other anatomical sites cannot be ruled out, since anti-Ly9 antibody therapy produced a marked, but not complete decrease of IL-10 levels.

Based on the anatomical location of MZB cells, they are one of the very first cells that come into contact with blood-borne pathogens and are therefore posed to mount the first line of the host defense response. It is intriguing how MZB cells would play a significant role in infections localized in the genital tract; however, it is possible that bacterial components, like cLPS, shedded to circulation would reach and activate splenic MZB cells. It is well-known that antigens trapped in the marginal zone activate MZB cells to rapidly become IgM secreting plasma cells or antigen presenting cells. The latter refers to MZB cells as inducers of effector adaptive immune responses against pathogens. In fact, it has been postulated that MZB cells play a protective role in *Staphylococcus aureus* and *Borrelia burgdorferi* infections (65). However, MZB cells have also been ascribed with a regulatory role of the immune response being the major source of IL-10 in *Listeria monocytogenes* and *Leishsmania donovani* infections (56, 66). Whereas, multiple cell types have been shown to be capable of producing IL-10, our results indicate that innate-like B cell are the most relevant IL-10 source after *C muridarum* stimulation. Furthermore, our results showed that the presence of IL-10-producing B cells is associated with decreased IFNγ T cell responses and impaired bacterial clearance ability. These effects would be probably mediated through the inhibitory effects of IL-10 on IFNγ production by NK cells, CD4^+^ T cells and CD8^+^ T cells (67, 68). Our findings suggest that, as described for the female tract infection, B cells also play a detrimental role in *C. muridarum* infection of the male genital tract. Innate activation and IL-10 production by B cells could be used by *Chlamydia* spp. as an immunoevasion strategy to establish chronic infections in susceptible hosts.

## Author Contributions

VR, RM, and AG: obtained funding and participated in study design; LS, GG, MG, FF, and MB: participated in study conduct, result interpretation, and drafting; VR and RM: participated in writing of the manuscript. All authors have contributed to the revision of the work, provided important intellectual content, and carefully reviewed and approved the final version of the manuscript. Authors agree to be accountable for all aspects of the work in terms of accuracy or integrity and other related aspects.

### Conflict of Interest Statement

The authors declare that the research was conducted in the absence of any commercial or financial relationships that could be construed as a potential conflict of interest.
